# Metabolic Remodelling: An Accomplice for New Therapeutic Strategies to Fight Lung Cancer

**DOI:** 10.3390/antiox8120603

**Published:** 2019-11-29

**Authors:** Cindy Mendes, Jacinta Serpa

**Affiliations:** 1 CEDOC, Chronic Diseases Research Centre, NOVA Medical School, Faculdade de Ciências Médicas, Universidade NOVA de Lisboa, Campo dos Mártires da Pátria, 130, 1169-056 Lisboa, Portugal; cindymendes8@gmail.com; 2 Instituto Português de Oncologia de Lisboa Francisco Gentil (IPOLFG), Rua Prof Lima Basto, 1099-023 Lisboa, Portugal

**Keywords:** lung cancer, cancer metabolism, reactive oxygen species (ROS), therapy resistance, new therapeutic strategies

## Abstract

Metabolic remodelling is a hallmark of cancer, however little has been unravelled in its role in chemoresistance, which is a major hurdle to cancer control. Lung cancer is a leading cause of death by cancer, mainly due to the diagnosis at an advanced stage and to the development of resistance to therapy. Targeted therapeutic agents combined with comprehensive drugs are commonly used to treat lung cancer. However, resistance mechanisms are difficult to avoid. In this review, we will address some of those therapeutic regimens, resistance mechanisms that are eventually developed by lung cancer cells, metabolic alterations that have already been described in lung cancer and putative new therapeutic strategies, and the integration of conventional drugs and genetic and metabolic-targeted therapies. The oxidative stress is pivotal in this whole network. A better understanding of cancer cell metabolism and molecular adaptations underlying resistance mechanisms will provide clues to design new therapeutic strategies, including the combination of chemotherapeutic and targeted agents, considering metabolic intervenients. As cancer cells undergo a constant metabolic adaptive drift, therapeutic regimens must constantly adapt.

## 1. Cancer Metabolism

Although cancer metabolism is one of the oldest areas of research in cancer biology, the study of metabolic alterations in tumours has grown exponentially in the past decade [1,2]. The link between cancer and metabolism was first made by Otto Warburg in 1923 with the observation that most cancer cells predominantly produce energy through a high rate of glycolysis followed by lactic acid fermentation, rather than through oxidative phosphorylation (OXPHOS) in the mitochondria [3]. Thus, tumours exhibit an increased rate of glucose uptake with lactate production, even in the presence of oxygen, through aerobic glycolysis with the production of carbon skeletons, NADPH and ATP [4]. This glycolytic switch, known as the “Warburg effect”, was first described as a compensation mechanism for mitochondria dysfunction in tumours. However, this view was challenged as several studies found that mitochondrial OXPHOS is active in most types of cancer [5,6,7,8]. To fulfil the biosynthetic demands associated with proliferation, mitochondria occupy a core status as providers of ATP and intermediate metabolites, such as citrate to supply anabolic reactions [9]. Moreover, in nontransformed cells, the Warburg effect is a reversible phenomenon linked to proliferation, showing that it reflects proliferation associated changes in metabolism instead of a unique feature of malignancy [10]. Glucose metabolism rewiring is more likely to be driven by an elevated demand of reducing equivalents and molecular precursors of proteins, nucleotides and lipids, which are the building blocks required to maintain cancer cell growth and proliferation [11]. Besides glucose, glutamine also contributes to core metabolic functions of cancer cells since it fuels the tricarboxylic acid (TCA) cycle, nucleotide and fatty acid biosynthesis and redox balance [10,12], replacing completely glucose as a core organic compound. 

Despite the fact that energetic metabolism is one of the cancer hallmarks, little has been shown in its role in chemoresistance [13]. Besides producing ATP, OXPHOS is the major source of reactive oxygen species (ROS) within mitochondria and in the entire cell [14]. During OXPHOS, the acetyl-CoA from glucose and fatty acids degradation feeds the TCA cycle, which generates reduced compounds [10,12] that will transfer electrons to the OXPHOS system through a chain of redox reactions with the final reduction of oxygen to water. In some of these reactions, ROS is a by-product [15], changing the mitochondrial membrane potential (MPP) and inducing damages in the respiratory chain, pushing cells towards apoptosis [14]. In response to augmented ROS, many tumours trigger protective antioxidant pathways [16]. Glutathione (GSH), peroxiredoxins, thioredoxin and superoxide dismutase (SOD2) belong to the major antioxidant system, maintaining the redox homeostasis [17,18,19]. Thus, the activation of those ROS scavenging processes constitutes a mechanism of resistance to chemotherapy exhibited by cancer cells, upon the exposure to alkylating or ROS generator drugs [14]. Changes in endogenous metabolism influence the metabolic course of xenobiotic compounds, which includes drugs used in cancer therapy. This fact reinforces the role of cancer metabolic rewiring as crucial in cancer cells survival and response to therapy. Here, we will discuss the main features of cancer metabolism and its influence on therapy resistance in lung cancer.

## 2. Metabolic Remodelling in Lung Cancer 

**Figure 1:**
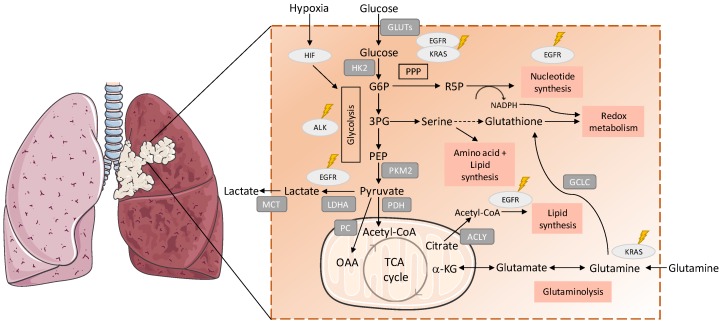
Metabolic remodelling in lung cancer. Metabolic pathways are involved in the synthesis of building blocks for macromolecules and redox homeostasis, needed for cell proliferation are presented. The import of glucose is mediated by the GLUT (glucose transporter) family of membrane transport proteins, which are known to be deregulated in cancer [34]. Hexokinase 2 (HK-2) is the first rate-limiting enzyme in the glycolytic pathway and overexpression of HK-2 has wildly been observed in cancer cells, correlating with poor overall survival in cancer patients [35,36,37]. Mutant EGFR (epidermal growth factor receptor 1) promotes metabolic remodelling in non-small-cell lung carcinoma (NSCLC) with increased aerobic glycolysis and PPP (pentose phosphate pathway), altered pyrimidine biosynthesis and upregulation of monounsaturated fatty acids [38,39,40]. *KRAS* (Kirsten rat sarcoma viral oncogene homolog GTPase)-mutated NSCLC cells express higher levels of enzymes involved in glycolysis, such as pyruvate kinase isozyme M2 (PKM2) and lactate dehydrogenase A (LDHA) compared with nonmalignant cells, indicating alterations in glucose metabolism and PPP (glicose-6-fosfato dehydrogenase (G6PD), transketolase (TKT) and 6-phosphogluconate dehydrogenase (6PGD)) [41]. Pyruvate is decarboxylated into acetyl-CoA to be further transported into mitochondria to enter the TCA cycle [42]. *ALK* (anaplastic lymphoma kinase) rearrangements were associated with upregulated glucose metabolism in highly metastatic phenotypes of adenocarcinoma [43]. The expression and activity of PC (pyruvate carboxylase), the enzyme responsible for the conversion of pyruvate into oxaloacetate, was found to be elevated in NSCLC tumours [28,33]. Glycolysis and glucose oxidation via PDH (pyruvate dehydrogenase) and the TCA cycle were enhanced in NSCLC comparing to adjacent benign lung [28]. Cancer cells also show higher levels of monocarboxylate transporters (MCT), which are responsible for lactate export and helps both in maintaining intracellular pH and in continuing glycolysis [44]. Hif-1 (hypoxia inducible factor 1) regulates the transcription of glycolytic enzymes such as, HK-2, LDH-A and PKM2, which upregulate glycolysis [45,46]. The expression of ATP citrate lyase (ACLY), a key enzyme in fatty acid synthesis was upregulated in NSCLC, being associated with poor prognosis [30]. Glutathione cysteine ligase (GCLC), which converts glutamate to Glutathione (GSH), is also highly expressed in several cancers, including lung cancer, and high mRNA expression of GCLC-promoted cisplatin resistance in lung adenocarcinoma cell lines [47]. G6P: glucose 6-phosphate; 3PG: 3-phosphoglyceric acid; PEP: phosphoenolpyruvate; R5P: ribose 5-phosphate; MCT: monocarboxylate transporters; OAA: oxaloacetate; α-KG: alpha ketoglutarate.

Lung cancer is a prevalent cause of cancer-related death [20], being grouped in two principal histological subtypes: small cell lung carcinoma (SCLC; 15% of total) and non-small-cell lung carcinoma (NSCLC; 85% of total) [21]. NSCLC is classified into three histotypes: squamous-cell carcinoma, adenocarcinoma, and large-cell carcinoma [22]. Metabolism was shown to be differently reprogrammed in the major subtypes of non-small cell lung cancer [23]. Metabolic remodelling is one of the emerging hallmarks of cancer [24] and it is well recognised that cancer cells have a high metabolic plasticity to support continuous cell growth and proliferation, meeting their energetic and biomass demands [25]. Recent studies reported metabolic alterations in glucose, lipids, amino acids and nucleic acids metabolism in NSCLC cells (Figure 1). 

In the past decade, stable-isotope tracing with ^13^C-glucose became an important tool for the analysis of metabolic pathways that are differentially activated in tumour cells in vivo, both in cancer mouse models and humans [26,27,28,29,30]. Uniformly labelled ^13^C-glucose is administered as a bolus by an intraoperative infusion before surgical tumour resection and the distribution of labelled carbons in the various intermediates is analysed by ^13^C NMR spectroscopy [31,32]. A study using fresh surgical resections from NSCLC patients with mixed histology, after a labelled ^13^C-glucose infusion, showed contrasting glucose metabolism results; tumour samples displayed high levels of lactate, demonstrating an upregulation in glycolysis, but also increased levels of glucose-derived TCA cycle intermediates, in tumour samples compared with normal tissue [30]. These observations reinforce the fact that glycolysis and OXPHOS can function in simultaneous if not in the same cancer cell at least in the same tumour, in which metabolic symbiosis can be established. Hensley and colleagues combined multimodal imaging analysis (FDG-PET and multiparametric MRI) and ^13^C-glucose flux profiling of NSCLC in situ to provide quantitative information about glucose metabolism and the tumour microenvironment in NSCLC untreated patients [28]. The activity of PC (pyruvate carboxylase), the enzyme responsible for the conversion of pyruvate into oxaloacetate, was elevated in NSCLC tumours [28,33], and its silencing significantly decreased the proliferative and colony-forming capacity of NSCLC cell lineages and reduced tumour growth in murine xenograft models, suggesting a dependence on PC-mediated and TCA cycle-based anaplerosis [33]. Moreover, it was found that glycolysis and glucose oxidation via PDH (pyruvate dehydrogenase) and the TCA cycle were higher in NSCLC compared to the adjacent normal lung [28]. Glucose-derived metabolic intermediates can be synthesized directly from glucose or indirectly from glucose-derived lactate. This fact was demonstrated by Faubert et al. [29]; lactate is the main carbon source for the TCA cycle in tumours from NSCLC patients and tumour xenografts. 

Several enzymes and transporters, crucial for carbon and energy metabolism, have been described as differently expressed in a normal lung and in cancer. The expression of ATP citrate lyase (ACLY), a key enzyme in fatty acid synthesis involved in the synthesis of acetyl-CoA and oxaloacetate, was upregulated in NSCLC, being associated with poor prognosis [48]. Glycine decarboxylase (GLDC), which couples decarboxylation of glycine to the biosynthesis of serine and one-carbon metabolism, takes part in pyrimidine metabolism and is upregulated in NSCLC cells [49]. xCT (SLC7A11), a cystine/glutamate antiporter, is overexpressed in the plasma membrane in NSCLC, correlating with patients’ worse survival [50]. Interestingly, it was described that cancer cells expressing high levels of xCT relied on glutamine and glutaminolysis dependency for OXPHOS [50]. This glutamine consumer phenotype may also be linked to cyst(e)ine dependency, as xCT concomitantly exports glutamate and imports cyst(e)ine. Glutamate is a direct product of glutamine degradation and maintaining the glutamine import sustains the import of cyst(e)ine, which has been related to increased therapy resistance in different cancer models [51], mostly due to its role in glutathione synthesis [52,53,54,55], for which glutamine-derived glutamate is also needed.

Despite similarities in metabolic reprogramming, the metabolic alterations in NSCLC cells or tumours are highly heterogeneous [28,39,56]. Hensley et al., identified metabolically heterogenous regions both within and between tumours, in which reduced perfusion tumour areas preferentially used glucose whereas well perfused regions relied more on non-glucose nutrients indicating that tumour metabolic remodelling is regulated by the microenvironment [28]. The generation of a hypoxic environment (oxygen deprivation) is a common feature of solid cancers as a consequence of the development of a disordered vasculature, which is not able to properly supply oxygen to a rapidly growing tumour [57]. Hypoxia is known to activate HIF signalling in cancer cells and plays an important role in the pathogenesis and prognosis of lung cancer [58]. Induction of HIF activity targets genes involved in glucose metabolism, angiogenesis, cell proliferation and resistance to therapies [59]. Furthermore, both Hif-1α and Hif-2α are frequently overexpressed in NSCLC correlating, in some cases, with poor prognosis [60,61] and Hif-1α expression is associated with resistance to cancer therapy, including EGFR (epidermal growth factor receptor 1) inhibitors in NSCLC [46].

Thus, understanding the influence of cellular or microenvironmental factors, such as oncogene-induced metabolic switches, on cancer cell metabolism is crucial for the development of better-adjusted therapeutic approaches targeting metabolic remodelling in cancer cells. 

### Role of Oncogenic Mutations (EGFR, ALK, KRAS) in Metabolic Remodelling

Certain genetic alterations have been shown as crucial in lung carcinogenesis. Mutations in *EGFR* (epidermal growth factor receptor 1) and *KRAS* (Kirsten rat sarcoma viral oncogene homolog GTPase) and *ALK* (anaplastic lymphoma kinase) rearrangements are mostly found in lung adenocarcinoma, accounting for 30–40% of NSCLCs [62]. Mutations in these oncogenes have been shown to play a role in metabolic reprogramming of cancer cells to support their high proliferative rate and energetic demands [39,63].

Mutant EGFR promotes metabolic remodelling in NSCLC with increased aerobic glycolysis and PPP (pentose phosphate pathway), altered pyrimidine biosynthesis and redox metabolism [38,39]. Treatment with erlotinib (EGFR inhibitor) and a glutaminase inhibitor (CB-839) generates a metabolic crisis in *EGFR* mutant NSCLC cells, resulting in cell death and in rapid tumour regression in mouse NSCLC xenografts [64]. These facts indicate the need of glutamine as a source for bioenergetics and biosynthesis in *EGFR*-mutated NSCLCs, as glucose is mainly used to sustain PPP and consequently cancer cells proliferation. Another study, showed the role of EGFR in the stabilization by phosphorylation of stearoyl-CoA desaturase-1 (SCD1), augmenting monounsaturated fatty acid synthesis and sustaining cell proliferation [40]. In addition, phosphorylated SCD1 levels were reported as an independent prognostic factor for poor survival in NSCLC [40]. 

The influence of *ALK* rearrangements on metabolism has not been well described in lung adenocarcinoma. However, a recent study observed the presence of upregulated glucose metabolism in highly metastatic phenotypes in this subset of lung cancer [43]. Again, increased consumption of glucose is linked to more aggressive cancer phenotypes.

Numerous studies showed the involvement of mutant KRAS in the metabolic rewiring of different types of cancer [63,65,66] with an upregulation of glucose uptake and aerobic glycolysis and increased glutamine utilization [67,68,69]. Proteomic profiles related to metabolism of intrinsic *KRAS* mutant NSCLC cell lines were investigated and compared with those of normal bronchial epithelial cells [41]. *KRAS*-mutated NSCLC cells expressed higher levels of enzymes involved in glycolysis (glyceraldehyde 3-phosphate dehydrogenase—GAPDH, pyruvate kinase isozyme M2—PKM2, lactate dehydrogenase A—LDHA and lactate dehydrogenase B—LDHB) and PPP (glicose-6-fosfato dehydrogenase—G6PD, transketolase—TKT and 6-phosphogluconate dehydrogenase—6PGD) compared with nonmalignant cells [41]. In another study, NSCLC cells carrying *KRAS* mutations showed metabolic remodelling with alterations in redox buffering systems and glutamine dependency [70]. Moreover, an upregulation of lactate production was observed in a mutant *KRAS* lung tumour mouse model [27], however, these mouse models minimally used glutamine as a carbon source for TCA cycle entry, while in the in vitro models, there was a dependence on glutamine. Thus, glutamine dependency can be related to the homeostatic cellular systems, such as GSH production, and not related to the direct and extensive used of glutamine as a carbon and energy source. In agreement with this, oxidative glucose metabolic enzymes, such as PC and PDH (pyruvate dehydrogenase), were shown to be necessary for tumour formation and growth in these mouse models [27]. Homozygous mutant KRAS cells have been shown to have an increased antioxidant capacity that accounts for their selective growth during lung tumour progression [63,71]. Varying oxygen levels within the growing tumour can contribute to this selection, since increased expression of Hif-2α promotes tumour growth and malignant progression of KRAS^G12D^ lung tumours [72]. These results correlated with existing human clinical data, implicating Hif-2α as a negative prognostic factor in human NSCLC [72].

## 3. Lung Cancer Therapy

Over the last 20 years, lung cancer treatment has evolved from the empiric use of cytotoxic therapies to effective and better tolerated targeted therapies. Platinum-based doublet therapy (combining platinum-based drugs with another cytotoxic/cytostatic agent) has been the standard therapy for both primary and palliative care of patients with advanced stage lung cancer [73,74]. Genotyping studies revealing genetic alterations in the various subtypes of lung cancer accounting for tumorigenesis led to the development of targeted therapies, namely directed to EGFR, ALK, and KRAS mutated variants [75]. 

In fact, the targeted therapy of patients with *EGFR*-mutated tumours is more effective than conventional therapy; and the efficacy of EGFR tyrosine kinase inhibitors (TKIs) is increasing over generations of drugs. First-generation EGFR inhibitors (e.g., gefitinib and erlotinib) have shown increased objective response (ORRs) and progression-free survival (PFS) compared to conventional cytotoxic treatment of patients suffering from EGFR-mutated tumours [73,76]. Second-generation inhibitors, such as afatinib and dacomitinib, are irreversible inhibitors that additionally target the receptors HER2 and HER4 (epidermal growth factor 2 and 4) and were reported to show higher PFS compared to first-generation EGFR inhibitors, such as gefitinib [77]. 

The fusion between echinoderm microtubule-associated protein-like 4 (*EML4*) gene and ALK (*EML4-ALK*) was the first fusion oncogene detected in lung cancer [78]. Fusion genes, involving ALK, are usually mutually exclusive with other oncogenic drivers such as EGFR and KRAS [79]. Crizotinib, a first-generation competitive ATP inhibitor of ALK tyrosine kinases with activity against ALK-fusion-positive NSCLC [80], is associated with higher ORRs and PFS in comparison to cytotoxic therapy in both conventionally treated and untreated patients [81]. In preclinical studies, ceritinib, a second-generation ALK inhibitor, has shown greater antitumour activity than first-generation inhibitors, as crizotinib [82]. Another fusion oncogene, encoding a constitutively activated tyrosine kinase, can result from fusion of ROS1 tyrosine kinase domain with CD74 [83]. Because it has a high homology with the kinase domain of ALK, ALK specific inhibitors, including crizotinib [84] and ceritinib [82], revealed marked activity in ROS1-positive tumours [85]. The same efficacy has been observed for crizotinib in NSCLC patients with tumours baring MET tyrosine kinase receptor amplifications [86,87].

Regarding *KRAS* oncogene, despite the fact that *KRAS*-MAPK pathway is downstream of EGFR signalling, *KRAS*-mutation-driven lung cancers, which are mostly adenocarcinomas, do not respond to EGFR TKIs [88] because the mutations in *KRAS* activate and release mutant KRAS from the upstream regulation. *KRAS* is the most frequently mutated oncogene in NSCLC patients, but effective therapies targeting mutant KRAS have yet to be developed. However, different therapies directed to KRAS downstream targets, such as MEK (MAPK/ERK kinase), are currently being tested. In a phase III study, NSCLC patients with *KRAS* mutated tumours treated with MEK inhibitor selumetinib plus docetaxel (taxane) did not show improved PFS compared to taxanes monotherapy [89]. Another MEK inhibitor, trametinib, was also evaluated alone or in combination with taxanes and revealed that combination with chemotherapy increased tolerability and clinical activity in both *KRAS*-mutant and *KRAS*-WT NSCLC patients [90]. 

Antiangiogenic therapy has also been tested in lung cancer patients. Bevacizumab, a monoclonal antibody against vascular endothelial growth factor (VEGF), in combination with paclitaxel (taxane) or carboplatin, significantly improved the median overall survival (OS) and PFS of NSCLC patients with tumours of nonsquamous cell histology [91]. 

Recently, immunotherapy has emerged as a potential treatment option against lung cancer, taking advantage of the native antitumour immune response [92]. Immune checkpoint blocker (ICB), generated upon T-cell activation, such as monoclonal antibodies that target cytotoxic T-lymphocyte antigen-4 (CTLA-4) and antibodies against PD-1 or PD-L1, are currently the most relevant targets for immunotherapy [73]. During tumorigenesis, PD-1 signalling inactivates T cells that recognize tumour-specific antigens, permitting tumour progression and metastasis [93]. ICBs, currently used or in development for NSCLC treatment, include the anti-PD-1 antibodies nivolumab (human IgG4) and pembrolizumab (humanized IgG4), along with the anti-PD-L1 antibodies atezolizumab (human IgG1, with the Fc domain engineered to prevent antibody-directed cell cytotoxicity), durvalumab (human IgG1 engineered), and avelumab (human IgG1 showing preclinical antibody-directed cell cytotoxicity activity) [74]. ICBs have been mostly used in patients with advanced NSCLC, whose tumours progress upon first-line cytotoxic therapy. Nivolumab treatment was associated with significantly longer median OS compared to treatment with docetaxel in patients with metastatic NSCLC, who had disease progression during or after platinum-based therapy [94,95]. The combination of anti-PD-(L)1 and anti-CTLA-4 monoclonal antibodies can result in higher and longer responses in NSCLC, as observed in experimental models and clinical studies [96,97]. Most patients, who achieve an initial benefit from an ICB eventually develop resistance, thus, the challenge is to develop rational combinations that will increase responses or delay the onset of resistance [98]. 

More recently, multiple trials have been investigating combinations of antiangiogenic agents and immunotherapy in NSCLC [99]. In particular, a clinical trial studied the efficiency of bevacizumab plus nivolumab in III/IV NSCLC patients and it was observed that the combinatory treatment improved PFS and decreased associated toxicity [100]. 

### 3.1. Mechanisms of Resistance to Conventional Therapy

Cancer chemotherapy resistance is one of the major dilemmas in cancer therapy, resulting in therapeutic failure and increased mortality. NSCLC cells are intrinsically resistant to various anticancer drugs, while SCLC cells can acquire resistance upon cyclic administration of a drug [101]. To address this issue, research has been focusing on how cancer cells modulate their genomes and metabolism to prevent drug influx, to facilitate efflux drugs, to inactivate drugs and/or to repair drug-induced damage [102]. More specifically, mechanisms of drug resistance identified so far include augmented drug eflux, drug inactivation and/or sequestration by enzymes, DNA repair, target modifications and apoptosis defects [101,103,104]. Ineffective drug delivery to the tumour, increased metabolism, lack of drug specificity to the tumour and tumour vasculature are additional contributing factors [105,106]. Additionally, patients treated with chemotherapy develop cumulative genetic mutations which may result in either activation of proto-oncogenes or inactivation of tumour-suppressor genes [101]. 

#### 3.1.1. Drug Transporters Increase the Efflux of Chemotherapeutic Drugs

The overexpression of ATP-binding cassette (ABC) membrane transporters leads to enhanced cytotoxic drug efflux and diminished intracellular accumulation, granting resistance to drugs such as cisplatin, methotrexate, taxanes, anthracyclines, and vinca alkaloids [101,107]. P-glycoprotein (P-gp) is codified by the multidrug-resistance (MDR-1) gene, belongs to the ABC superfamily and functions as an energy-dependent efflux pump of metabolites [108]. Studies have shown an increased expression of P-gp in lung tumours, with notably higher rates in NSCLCs than in SCLCs [101]. In addition, major vault protein (MVP), also known as the lung resistance-related protein (LRP), has been pointed out to be involved in lung cancer drug resistance [109]. 

#### 3.1.2. Drug Inactivation by Sulphur-Containing Molecules and Role of Antioxidants as a Cause of Drug Resistance

Another mechanism of resistance is by conjugation of the drug with sulphur-containing macromolecules such as metallothioneins (MTs) and GSH [101,110]. MTs are intracellular proteins rich in cysteine content (30%) that bind to cytotoxic agents such as platinum compounds and alkylating agents [111]. High MTs levels have been observed in tumour cells with acquired resistance to alkylating agents [110,112]. Moreover, augmented expression of MTs was described in NSCLC with squamous cell lung carcinoma and adenocarcinoma histotypes, but it was not demonstrated in SCLC [113]. Studies also show that MTs play a relevant role in the cellular protection against oxidative stress [114]. A strong correlation between MTs expression and cisplatin and doxorubicin resistance was observed in different cell lines of SCLC [115,116]. The GSH S-transferases (GSTs) protect cancer cells from reactive endogenous and exogenous electrophiles, such as prostaglandins, aromatic hydrocarbons and chemotherapeutic agents, through the conjugation with GSH (the most abundant cellular thiol), and scavenges them [101,110,117]. In tumour cells, expression levels of GSTs are increased in comparison to normal cells which may contribute to elevated detoxification of anticancer drugs [118,119]. GST isoenzymes have been reported in lung tumours in higher levels than in normal bronchioles and alveoli [120,121].

More than one mechanism of resistance can act on the same cancer cell/tumour. GS-conjugates are transported out of the cells by efflux transporters, such as multidrug resistance protein 1 (MRP1) and P-gp, thus conferring increased levels of resistance to the cytotoxicity of antineoplastic drugs [122,123]. Multiple studies involving NSCLC and SCLC cell lines suggested that high levels of GSH were associated with decreased platinum-DNA binding and intracellular platinum accumulation, increasing cisplatin resistance [124,125,126]. Conversely, factors inducing the reduction of cellular GSH content sensitise cancer cells to cisplatin [127]. Nuclear factor erythroid-like 2 (NRF2), a regulator of redox homeostasis upon oxidative stress, is activated by cells in order to upregulate gene networks involved in cytoprotective activities [128]. This transcription factor has been shown to be upregulated in various types of cancer, including skin, breast, prostate, lung and pancreas [129] and has also been associated with chemoresistance [130]. xCT, a downstream target gene of NRF2, responsible for the import of cysteine to support GSH synthesis has been implicated in multidrug resistance of lung cancer [131]. In particular, the x_c_^–^ amino acid transport system maintained intracellular GSH and consequently resulted in cisplatin resistance in ovarian cancer cells [132]. 

Furthermore, cancer cells may develop resistance by overexpressing antioxidants, which protect cells from chemotherapy-induced oxidative stress and cell death, consequently a new redox balance with higher ROS levels is established, a process called “redox resetting” [101,133]. Epirubicin is known to cause oxidative stress by the generation of superoxide and hydrogen peroxide (H_2_O_2_) moieties, which drive cancer cell death [134]. Nevertheless, overexpression of antioxidants (e.g., SOD or GSH) neutralizes the oxidative stress leading to drug resistance. Accordingly, high levels of manganese SOD (MnSOD) seems to protect lung epithelial cells against oxidative injury [135]. Moreover, malignant mesothelioma cells were reported to have higher levels of SOD mRNA and activities compared with nonmalignant mesothelial cells, but also had elevated catalase and GSH levels, being more resistant to H_2_O_2_ and epirubicin [136]. Accordingly, platinum drugs that generate very high ROS levels can be inactivated by GSH [137].

Alterations in drug metabolism are also associated to resistance since they can lead to drug inactivation or deficient drug activation. Antioxidant systems are able to directly inhibit the antitumour activity of several anticancer agents, such as paclitaxel [138], bortezomib [139] and radiation therapy [140]. Buthionine sulphoximine (BSO) significantly increases paclitaxel cytotoxicity through ROS accumulation [138]. The cellular redox state is associated with enzymatic expression required for the conversion of antimetabolites including 5-fluorouracil (5-FU) and methotrexate to their most active forms [133,141]. Capecitabine is an anticancer agent that is converted into 5-FU by thymidine phosphorylase [142], which is encoded by the *TYMP* gene that can be inactivated by DNA methylation causing capecitabine resistance [143]. These epigenetic alterations can be induced by H_2_O_2_ as DNA methyltransferase 1 (DNMT1) binds more strongly to chromatin after H_2_O_2_ exposure altering the methylation status of CpG regions [144]. The inactivation by UDP glucuronosyl transferase 1 (UGT1A1) by the topoisomerase inhibitor, irinotecan, is induced by the redox-sensing NRF2-KEAP1 pathway [145]. On another hand, the expression of UGT1A1 is decreased by promoter DNA methylation, promoting irinotecan activity [146].

#### 3.1.3. DNA-Repair Pathways Inducing Resistance to Chemotherapy

As DNA damage is the main objective of most chemotherapeutic agents, increased capacity of DNA damage repair is one possible mechanism of resistance to the cytotoxic effects of anticancer drugs. Cisplatin for instance induces apoptosis by forming DNA-platinum adducts and by generating ROS, which causes oxidative DNA damage [147]. The nucleotide excision-repair (NER) pathway is one DNA repair pathway involved in the acquisition of platinum-based drug resistance [101,148]. Excision-repair cross-complementation group 1 (ERCC1) protein is a molecular indicator of resistance to platinum salts and forms the molecular complex of the NER pathway along with other proteins that are able to correct nucleotides modified by DNA-platinum adducts [149]. An association was found in in vitro studies between the expression of ERCC1 mRNA in NSCLC and resistance to platinum drugs, showing that a low expression of ERCC1 correlated with prolonged survival of NSCLC patients, who were treated with cisplatin plus gemcitabine [150,151]. Mismatch-repair (MMR) pathway repairs base-base and insertion-deletion mismatches during DNA replication [152]. This pathway can repair DNA-platinum adducts, which often results in mitotic stress and cell death [153]. However, this repair pathway is not considered relevant as a mechanism of chemotherapy resistance in lung cancer [101]. In contrast, base-excision-repair (BER) pathway was correlated with chemoresistance, proved by the fact that N-methylpurine DNA Glycosylase (MPG) and Apurinic/Apyrimidinic Endonuclease (APE) inhibition or elimination lead to increased resistance to alkylating agents, such as platinum-based drugs [154]. 

#### 3.1.4. Loss of Intracellular Commands of Cell Death as a Cause of Drug Resistance

Cell death inhibition is another way of contributing to drug resistance. Failure of the intracellular death signalling pathways lead to the alteration of various apoptotic and antiapoptotic intracellular proteins, including Bcl-2, Bax and SAPK/JNK in several types of cancer [101,155]. Indeed, in vitro and in vivo evidence have shown that overexpression of Bcl-2 in SCLC contributes to apoptosis resistance [156]. Cancer cells with defective caspases are resistant to drugs, whose main mechanism of action is the induction of apoptosis. In this sense, NSCLC cells showing low expression of caspases 3 and 9 are resistant to cisplatin chemotherapy [157]. Alterations in antiapoptotic proteins such as IAP (inhibitor of apoptosis protein), particularly XIAP (X-linked inhibitor of apoptosis protein), and survivin have been observed in NSCLC patients with implications in resistance. It was shown that XIAP was overexpressed in human H460 NSCLC cell line, leading to the inhibition of the apoptosome formation, pivotal in caspase-dependent cell death [158]. Low expression of survivin in patients was associated with a significantly better OS in comparison to patients with tumours displaying high expression of this protein [159]. 

Drugs inducing cell death by affecting microtubule stability, such as paclitaxel, kill cells in a Fas/Fas ligand (FasL)-dependent manner [160]. Blockade of Fas/FasL and inhibition of FasL expression by Bcl-2 overexpression are resistance mechanisms to paclitaxel [160]. Conversely, phosphorylation of Bcl-2 induces the expression of FasL, mediated by the nuclear action of NFAT (nuclear factor of activated T lymphocytes), which is responsive to microtubule damage, thereby restoring paclitaxel sensitivity [161].

### 3.2. Mechanisms of Resistance to Targeted Therapy

Although targeted therapies are revolutionizing the treatment of advanced NSCLC, resistance appears in most patients sooner or later [162]. In this section, we review the mechanisms of resistance that have been discovered in the past few years, in particular to TKIs directed to EGFR, ALK and ROS1, and later we will discuss strategies to overcome drug resistance. The first-generation erlotinib and gefitinib, and second-generation afatinib are now recognized as the standard first-line therapy in NSCLC patients with activating EGFR mutations [76,163,164]. However, some patients do not respond to EGFR-TKIs (intrinsic resistance). The most common mechanism for the acquired resistance to EGFR-TKIs is the development of the T790M second mutation within the *EGFR* kinase domain, which contributes for 50% of all acquired resistance [165]. The methionine 790 sterically blocks its interaction with TKIs, increasing affinity for ATP and reducing binding of the inhibitor to the kinase domain of EGFR, while keeping the catalytic activity [166]. The *MET* gene amplification is another frequent mechanism of acquired resistance and affects 5–20% EGFR-TKI treated NSCLC patients, irrespective of the T790M mutation status [167,168,169]. Although *HER2* amplification is a rare event in lung adenocarcinoma, it accounts for about 1–2% of total cases and up to 13% of NSCLC with acquired resistance to EGFR-TKIs [170,171]. Mutated EGFR heterodimerizes with HER2 resulting in heterodimers resistant to degradation [171], supporting EGFR-TKIs resistance in presence of both T790M mutation and *HER2* amplification itself as an acquired mechanism of drug exhaustion. *KRAS* and *EGFR* mutations are usually mutually exclusive but when they coexist, mainly in tumours under EGFR-TKIs treatment, *KRAS* mutations can confer resistance to EGFR inhibitors [172]. A study including 60 lung adenocarcinomas, either refractory or sensitive to both gefitinib and erlotinib, indicated that *KRAS* mutations lead to a lack of sensitivity to these drugs [172]. 

Another important pathway that has been associated to resistance to EGFR therapy is the PTEN-PI3K-AKT pathway. Phosphatase and tensin homolog (PTEN) acts as a tumour suppressor gene that can decrease tumour growth by inhibiting Akt [173], which can promote cell survival by inactivating several apoptosis mediators, such as Bad and caspases-9. Thus, loss of PTEN leads to increased tumours, since PTEN regulates negatively the PI3K-AKT pathway [174]. In EGFR mutant lung cancer, loss of PTEN led to resistance to EGFR inhibitors such as erlotinib [175], because PI3K activation can somehow interconnect PI3K-AKT and MAPK pathways. 

Loss of E-cadherin expression and upregulation of mesenchymal proteins, including vimentin, fibronectin and N-cadherin, are the main characteristic of epithelial–mesenchymal transition (EMT). AXL (AXL receptor tyrosine kinase) is associated with EMT in several tumours and its upregulation in the Hedgehog pathway has been recognized as a mechanism of resistance to targeted drugs in EGFR-mutated NSCLC [176]. In a recent study, gefitinib-mediated ROS promoted EMT and mitochondrial dysfunction concomitant with resistance of lung cancer cells [177]. Moreover, gefitinib treatment in the presence of ROS scavenger provided a partial rescue of mitochondrial aberrations, suggesting that antioxidants may alleviate ROS-mediated resistance. 

The evolution of a same tumour from adenocarcinoma to squamous cell carcinoma along the administration of anti-EGFR drugs is a mechanism of acquired drug resistance [178]. However, the cause of resistance to anti-EGFR targeted therapy in 18–30% of NSCLC patients still remains unknown [167,170]. The occurrence of tertiary EGFR mutations has been frequently reported in cases with acquired resistance to third-generation TKIs, being demonstrated in in vitro models [179]. Resistance to osimertinib is mainly caused by the EGFR p.Cys797Ser (C797S) mutation in exon 20, consisting of a substitution of a cysteine to a serine in the tyrosine kinase domain, decreases the action of third-generation TKIs, since it reduces their covalent binding to EGFR [180]. The presence of triple mutants (sensitizing mutation, T790M and C797S) supports the resistance to all three generations of EGFR TKIs [181]. 

NSCLC cells resistant to EGFR TKIs, gefitinib and erlotinib, were shown to exhibit elevated OXPHOS accompanied by elevated glycolysis and activity in TCA cycle [182]. In A549 NSCLC cell line, erlotinib drove ROS-mediated apoptosis via activation of the c-Jun N-terminal kinase (JNK) pathway, leading ultimately to EGFR inhibition [183]. Administration of the ROS scavenger N-acetyl cysteine reversed this phenomenon [184]. 

Tumours driven by either *ALK* or *ROS1* involving fusion genes exhibit similar mechanisms of resistance to targeted agents. It is well acknowledged in *ALK* mutated lung cancer the occurrence of mutations in the tyrosine kinase domain, for example L1196M and C1156Y, upon treatment with crizotinib [185]. The L1196 and G1269A substitutions are among the most frequently reported single-nucleotide mutations causing crizotinib resistance in NSCLC [186]. The existence of *ALK* or *ROS1* rearrangements together with *KRAS* mutations in NSCLC may explain primary or acquired resistance to crizotinib [187,188]. Accordingly, KRAS and NRAS activation through mutations promotes the exhaustion of first-generation inhibitors activity in ROS-1 positive cellular models [189]. Relatively to patients treated with second-generation TKIs, the most common *ALK* resistance mutation is G1202R, which is associated with in vitro resistance to all currently available ALK inhibitors excluding lorlatinib, a third-generation ALK inhibitor that shows great efficacy in patients with ALK resistance mutations [190], which are more common after treatment with second-generation ALK inhibitors. After ceritinib and alectinib treatment, missense mutations were observed in more than 50% of the samples, compared with the 30% of target alterations responsible for crizotinib exhaustion [186]. Despite the efficacy of crizotinib is thought to be specific to ALK inhibition, crizotinib also acts via the generation of superoxide and induction of apoptosis [191].

Resistance against angiogenesis inhibitor bevacizumab was also reported [192]. VEGF binds not only to its tyrosine kinase receptors (VEGFR), which can also interact to Neuropilin-1 (NP1) and Neuropilin-2 (NP2) [193]. Co-expression of NP1 and NP2 in NSCLC tissues is significantly correlated with tumour progression and bad prognosis [194]. Given that bevacizumab blocks VEGF-A, NP1 and NP2 under other stimuli may still increase the effects of VEGFR-1 and VEGFR-2, promoting angiogenesis and activating alternative pathways. 

## 4. Metabolic Remodelling in Lung Cancer in Response to Oxidative/Alkylating Treatment

As mentioned above, cisplatin interacts with reducing equivalents, such as GSH and DNA, accounting for increased ROS and DNA damage, which leads to apoptosis [195]. Different studies suggest that metabolic remodelling, in cisplatin-resistant lung cancer cells, involved redox buffering to abrogate cisplatin effect [196,197,198,199]. Those lung cancer cells display higher levels of ROS, in part related to the low levels of intracellular thioredoxin [198], but also due to the high levels of GSH and GCL-C (glutamate cysteine ligase catalytic subunit), the first enzyme acting on the synthesis of GSH [195], possibly to counteract the high ROS levels induced by cisplatin [200]. Several studies showed that cisplatin-resistant cells are vulnerable to rapidly ROS-inducing agents. Indeed, a study reported that cisplatin-resistant lung cancer cells were more sensitive to elesclomol, an agent known to exponentially augment ROS [197] inside the cell in such a fast way that cancer cells do not have means of adapting. The xCT cyst(e)ine/glutamate pump, which supplies cells with cysteine essential for GSH production, is upregulated in cisplatin-resistant cells, as they are more sensitive to the xCT inhibitor riluzole as compared to their parental nonresistant cells [197]. Additionally, cisplatin-resistant lung cancer cells have lower rates of glycolysis and rather rely on OXPHOS [201]. Lower levels of hexokinase 1 (HK1) and 2 (HK2), the enzymes that catalyse the first step of glycolysis [202], were also observed in these lung cancer cells, in agreement with the fact that cisplatin exposure decreases HK expression [201,203]. Cisplatin-resistant lung cancer cells also showed decreased levels of glycolysis and lactate production in comparison to the sensitive parental cell lines [197] indicating a lower glycolytic activity or an increased OXPHOS. Under normal growth conditions, cisplatin-resistant lung cancer cells are not sensitive to glucose starvation, however, under hypoxic conditions, these cells are more vulnerable for 2-deoxyglucose (2-DG) treatment, a competitive inhibitor of HK, as compared to the parental cells [196]. Since cells depend on glycolysis for their energy production in the absence of oxygen, the reduced levels of HK in cisplatin-resistant cells probably makes them more vulnerable for 2-DG [201]. Greater rates of OXPHOS and mitochondrial activity, as well as a higher dependence on glutamine are observed in cisplatin-resistant lung cancer cells to compensate the lower glycolytic activity [197,198,201]. Furthermore, to fuel the TCA-cycle, β-oxidation of fatty acids has been reported in cisplatin-resistant lung cancer cells [201]. A recent study showed that cisplatin-resistant lung adenocarcinoma cells have higher MMP and intracellular ATP levels than the nonresistant cells, which also confer them increased aggressiveness [204]. 

## 5. New Therapies, a Comprehensive Adjustment of Therapy to the Metabolic Remodelling

**Figure 2:**
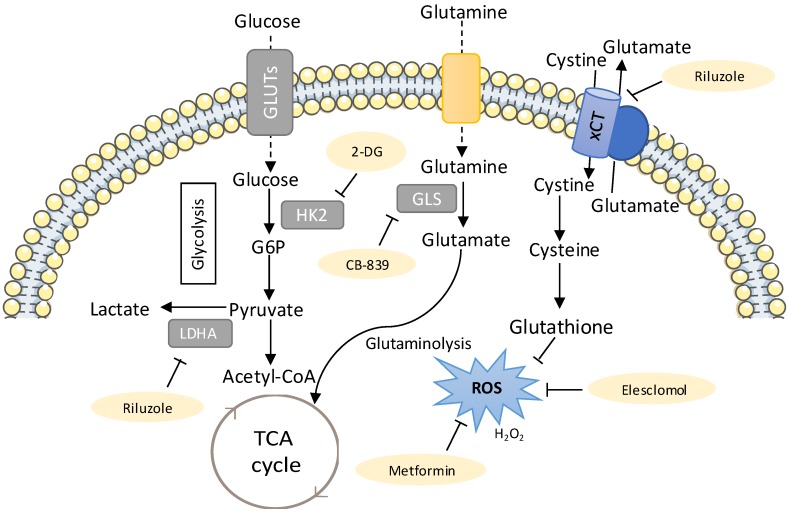
New metabolic therapies targeting drug-resistant lung cancer. The metabolic inhibitors that can be used to target drug-resistant cancers are shown in yellow. The sugar analogue 2-deoxyglucose (2-DG, competitive inhibitor of Hexokinase (HK), has been shown to be selectively cytotoxic to several tumour cell lines under anaerobic and/or hypoxic conditions [208,209] and to reduce resistance to cisplatin in lung cancer cells and an in vivo xenograft model of lung cancer [201]. Thus, 2-DG has the potential of being used clinically as an adjuvant to the classical chemotherapeutic compounds, such as cisplatin. Riluzole interferes with glutamate flux and was shown to increase reactive oxygen species (ROS) by suppressing lactate dehydrogenase A (LDHA) and NAD+ [197]. Glutamate stimulates the glutamine import and glutaminolysis, sustaining the import of cyst(e)ine, which has been related to increased therapy resistance in different cancer models [51], mostly due to its role in glutathione synthesis [52,54]. Riluzole interferes with system xCT-cystine/glutamate antiporter, resulting in decreased GSH levels [197]. Thus, using riluzole as an antitumour agent against cisplatin resistance in lung cancer patients could be further explored. Treatment with CB-389, a glutaminase inhibitor generated a metabolic crisis in EGFR mutant NSCLC cells, resulting in cell death and in rapid tumour regression in mouse NSCLC xenografts [64]. Metformin, an antidiabetic drug, was observed to increase the sensitivity of carboplatin-resistant NSCLC cells to carboplatin treatment in vitro and in vivo [210] and also reversed resistance to tyrosine kinase inhibitors (TKIs) and ALK inhibitors in lung cancer [211]. Another study demonstrated that metformin enhanced the sensitivity to a combined treatment of cisplatin and ionizing radiation in H460 and A549 NSCLC cell lines, with a greater effect in the A549 cell line, which is less sensitized by cisplatin [212]. Elesclomol is another potential therapeutic agent, since it further increased ROS in cisplatin-resistant cells, pushing them beyond their tolerance limit, which ultimately leads to cell death [199].

Anticancer drug resistance is often linked to metabolic alterations and these may be targeted to overcome this issue (Figure 2). Riluzole, a FDA-approved drug for the treatment of amyotrophic lateral sclerosis [205], interferes with glutamate flux and blocks metabotropic glutamate receptors (GRM) signalling. This drug blocked proliferation of melanoma cells expressing GRM in vitro, in vivo and in a phase-0 trial, making riluzole a promising drug to treat melanoma [206,207]. In cisplatin-resistant lung cancer cells, treatment of riluzole disrupted the oxidative defense by significantly reducing glutamate release which, in turn, suppressed GSH levels, resulting in higher ROS accumulation. Riluzole treatment increased ROS by suppressing lactate dehydrogenase A (LDHA) and NAD^+^ levels and blocked the cystine/glutamate pump, leading to cell death in cisplatin-resistant cells [197]. Therefore, using riluzole as an antitumour agent against cisplatin resistance in lung cancer should be further explored. 

The sugar analogue 2-DG has been shown to be selectively cytotoxic to several tumour cell lines when cultured under anaerobic and/or hypoxic conditions [208,209] and to reduce resistance to cisplatin in an in vivo xenograft model of lung cancer [201]. Another clinical trial reported that 2-DG in combination with docetaxel was well tolerated [213]. Under hypoxia, 2-DG and 2-fluorodeoxyglucose (2-FDG) treatment inhibited glycolysis, and thus lactate production, and also induced higher cell death in cisplatin-resistant cells with low levels of HK2, as compared to their respective parental cells [201]. Hence, targeting metabolic pathways using glycolytic inhibitors, such as 2-FDG or 2-DG, to kill cisplatin-resistant lung cancer cells under anaerobic/hypoxic conditions can be an interesting therapeutic approach. 

The ability to repair cisplatin-DNA adducts appears to be involved in the development of cisplatin resistance. The nucleotide excision-repair (NER) pathway and the *ERCC1* gene have been pointed out as attractive molecular targets to increase the cytotoxic effects of platinum compounds and overcome their resistance [214]. Metformin has been used for more than 50 years for the treatment of type 2 diabetes mellitus [215] and several studies showed that it has anticancer properties, improving the prognosis of patients with multiple cancers and decreasing the risk of cancer development [216,217]. A study demonstrated that metformin enhanced the sensitivity to a combined treatment of cisplatin and ionizing radiation in in vitro NSCLC models, with a greater effect in cells that are less sensitive to cisplatin [212]. These authors also showed a significant reduction in the expression of *ERCC1* after metformin treatment, pointing a possible involvement of the NER pathway in the radio-enhancement effect of the combined cisplatin and metformin treatment. Moreover, metformin was found to reverse resistance to TKIs and ALK inhibitors in lung cancer [211]. Another study using metformin showed that it increased the sensitivity of carboplatin-resistant NSCLC cells to carboplatin treatment in in vitro and in vivo models [210]. Metformin treatment decreased the expression of pyruvate kinase muscle isozyme M2 (PKM2), the enzyme that catalyses the final step in glycolysis, and consequently inhibited partially glucose metabolism and reduced ATP levels in carboplatin-resistant NSCLC cells [210]. 

Tumour cells have the ability to adapt their metabolism to different environments and stressful conditions, increasing adaptability and tumour response to therapies [13,15]. The control of the mitochondrial biogenesis can be a mean of adaption, which is preferentially regulated by the peroxisome proliferator-activated receptor gamma (PPAR-γ) transcriptional coactivator-1 alpha (PGC-1α) [218]. During the OXPHOS process, protons are pumped into the mitochondrial inner membrane potential (MIMP), which is finally dissipated through Complex V, generating ATP [219]. In a study using cisplatin-resistant NSCLC cells, two out of three cell lines showed stable changes towards an augmented OXPHOS function and decreased glycolysis [220]. However, the three cell lines responded in a similar way increasing ROS, MIMP and mitochondrial mass as an early response to cisplatin treatment. The authors also observed a decrease in AIF (proapoptosis) and an increase in Bcl2 (anti-apoptosis), indicating that this mechanism does not replace other classical mechanisms of cisplatin resistance [220]. A stable increase of PGC-1α, is seen in cells with increased OXPHOS activity. Treatment with the mitochondrial inhibitors metformin or rotenone (inhibitors of the complex I of the OXPHOS system) reduces the viability of the cell lines proportionally to their OXPHOS requirements [220]. This study provides new insights into cisplatin resistance mechanism in NSCLC cells which may lead to the design of new therapeutic approaches targeting mitochondria. 

In NSCLC, Yuan et al., suggested that PKM2 knockdown could serve as a chemosensitizer to docetaxel, leading to the inhibition of cell viability, cell cycle arrest at G2/M phase and apoptosis [221]. These results further suggest that application of targeting the PKM2 has the potential to be a therapeutic strategy for NSCLC and provides one possible way to improve the chemotherapy effect of docetaxel. The use of NRF2-targeting agents to overcome this chemoresistance has been studied extensively. Stable knockdown of either NRF2 or KEAP1 in NSCLC cells resulted in sensitization to chemotherapeutic drugs. In particular, silencing of KEAPI augmented the expression of PPARγ and genes associated with differentiation [222]. Another study using a mouse model of mutant KrasG12D-induced lung cancer showed that suppressing the NRF2 pathway with the chemical inhibitor brusatol enhanced the antitumour efficacy of cisplatin and reduced the tumour burden as well as improving survival [223]. 

Although long-term gefitinib treatment can provide effective action against its primary target (aberrant EGFR activity), secondary effects result in high generation of ROS [177]. In a study using lung adenocarcinoma cells, gefitinib treatment, in the presence of a ROS scavenger, provided a partial rescue of mitochondrial aberrations. In addition, the withdrawal of gefitinib from a priori resistant clones correlated with normalized expression of EMT genes. These findings suggest that antioxidants potentially provide therapeutic benefits by attenuating TKI-induced ROS and EMT [177]. 

In a recent study by Apicella et al., lactate metabolism was found to be involved in the resistance to MET and EGFR TKIs (JNJ-605, crizotinib and erlotinib, respectively), in which patient-derived NSCLC showed upregulated glycolytic metabolism, with high release of lactate [224]. Lactate can act as a signaling molecule which instructs cancer associated fibroblasts (CAFs) to produce hepatocyte growth factor (HGF), whose secretion activates MET signaling in cancer cells, overcoming TKI inhibitory effects [31,224]. The pharmacological inhibition of LDH, MCT4 and MCT1 was sufficient to abrogate the in vivo resistance, making these inhibitors a new therapeutic approach that simultaneously targets lactate metabolism and oncogenes to overcome targeted therapy resistance [224].

## 6. Conclusions

Improved understanding of both cellular metabolism and resistance mechanisms at the molecular level promotes new opportunities to combine chemotherapeutic agents with targeted agents, which may be a promising strategy to overcome chemoresistance and to increase the effectiveness of therapy for lung cancer patients. A concomitant challenge is to find the exact killing profile and adapt it to the metabolic drift in which cancer cells continuously undergo. The course of metabolic adaptation to stressful conditions, such as drug exposure, brings together specialized tumour cell characteristics that are specific to the tumour and the individual. In the future, precision medicine will have to combine metabolic monitoring and evolution data in order to adapt clinical regimens and take advantage of the weaknesses of cancer for therapeutic purposes.
